# CDH1 somatic alterations in Mexican patients with diffuse and mixed sporadic gastric cancer

**DOI:** 10.1186/s12885-019-5294-0

**Published:** 2019-01-14

**Authors:** Andrea Rebeca Bustos-Carpinteyro, Carla Oliveira, Abel Sousa, Patricia Oliveira, Hugo Pinheiro, Joana Carvalho, María Teresa Magaña-Torres, María Guadalupe Flores-Miramontes, Adriana Aguilar-Lemarroy, Luis Felipe Jave-Suárez, Jorge Peregrina-Sandoval, José Alfonso Cruz-Ramos, Josefina Yoaly Sánchez-López

**Affiliations:** 1 División de Genética, Centro de Investigación Biomédica de Occidente, Instituto Mexicano del Seguro Social, Sierra Mojada N. 800, Col. Independencia, C. P. 44340 Guadalajara, Jalisco México; 2 Doctorado en Genética Humana, Universidad de Guadalajara, Guadalajara, Jalisco México; 3 Expression Regulation in Cancer, IPATIMUP, Institute of Molecular Pathology and Immunology of the University of Porto, Porto, Portugal; 4 Instituto de Investigação e Inovação em Saúde (i3S; University of Porto, Porto, Portugal; 5 Faculty of Medicine, University of Porto, Porto, Portugal; 6 Department of Internal Medicine, Centro Hospitalar Tâmega e Sousa Avenida do Hospital Padre Américo, N° 210 4564-007, Guilhufe - Penafiel, Portugal; 7 División de Inmunología, Centro de Investigación Biomédica de Occidente, Instituto Mexicano del Seguro Social, Guadalajara, Jalisco México; 8 Laboratorio de Inmunobiología, Departamento de Biología Celular y Molecular. Centro Universitario de Ciencias Biológicas y Agropecuarias, Universidad de Guadalajara, CP 45510 Nextipac, Jalisco México; 9 Instituto Jalisciense de Cancerología, CP 45180 Guadalajara, Jalisco México

**Keywords:** DNA sequencing, *CDH1* mutations, LOH, Methylation, Gastric Cancer

## Abstract

**Background:**

Diffuse gastric cancer (DGC) is associated with the reduction or absence of the expression of the cell adhesion protein E-cadherin (encoded by the *CDH1* gene). Molecular characteristics are less well described for mixed gastric cancer (MGC). The main somatic alterations that have been described in the *CDH1* gene are mutations, loss of heterozygosity (LOH) and promoter methylation. The aim was to analyze *CDH1* somatic alterations in Mexican patients with diffuse and mixed gastric cancer.

**Methods:**

We searched for mutations in the *CDH1* gene in tumor DNA from DGC (*n* = 13) and MGC (*n* = 7) patients by next generation sequencing (NGS). Validation of findings was performed using Sanger sequencing. LOH was analyzed using dinucleotide repeat markers surrounding the *CDH1* gene, and methylation was investigated by DNA bisulfite conversion and sequencing. E-cadherin protein deficiency was analyzed by immunohistochemistry.

**Results:**

Seventeen point variants were identified by NGS, 13 of them were validated by Sanger sequencing. Only 1/13 had not been previously reported (c.-137C > A), and 12/13 were already reported as polymorphisms. Two DGC cases presented LOH at the locus 16q22.1 (13.3%). *CDH1* promoter methylation was positive in (7/11) 63.6% and (4/6) 66.6% of the cases with DGC and MGC, respectively. E-cadherin protein deficiency was observed in 58.3% of DGC cases while 100% in MGC cases.

**Conclusions:**

While no pathogenic somatic mutations were found that could explain the diffuse histology of gastric cancer in DGC and MGC, methylation was the most common somatic inactivation event of the *CDH1* gene, and LOH was rare. The previously unreported c.-137C > A variant modify the *CDH1* gene expression since it alters the binding sites for transcription factors.

**Electronic supplementary material:**

The online version of this article (10.1186/s12885-019-5294-0) contains supplementary material, which is available to authorized users.

## Background

Gastric cancer (GC) represents the fifth most frequent cancer type worldwide, with developing countries accounting for 70% of the cases. In terms of mortality, GC ranks third after lung and liver cancers [1]. The GC mortality represents 8.0% of total cancer deaths, with a rate of 5.5/100,000 inhabitants [1]. Despite a worldwide reduction in the prevalence of GC in recent years, this is not the case in Mexico where it remained constant. Several risk factors are implicated in the development of GC and the main biological-infectious factor is the bacteria *Helicobacter pylori* [2].

Histologically, GC can be divided into diffuse (DGC), intestinal and mixed (MGC) or indeterminate according the Lauren classification [3]. It has been observed that clinicopathological features and survival rates are related to the genetic/epigenetic alterations present in the tumors [4]. Genetically, DGC has been mainly associated with changes in the *CDH1* gene*,* which encodes the E-cadherin protein [5]. E-cadherin is a cell-cell adhesion protein crucial for maintaining the structure and function of epithelial tissues [6]. In GC, a reduced or null E-cadherin expression has been correlated with infiltrative capacity and metastasis; in this type of cancer, the most commonly reported anomalies in E-cadherin are germline and somatic mutations, and somatic loss of heterozygosity (LOH) and promoter methylation [6].

A recent study describing *CDH1* somatic alterations in GC reported a mutation frequency of 4.5%, LOH frequency of 4.5% and methylation frequency of 25.4% in diffuse and mixed gastric tumors [4]. Of notice is the fact that structural alterations (mutations and LOH) were associated with worse prognosis [4]. Due to the important role of *CDH1* alterations in gastric cancer, the aim of this study was to analyze *CDH1* somatic alterations (mutations, LOH and methylation) in Mexican patients with DGC and MGC.

## Methods

### Patients and samples

A total of 20 samples of fresh gastric biopsies with a histologic diagnosis of diffuse (*n* = 13) or mixed (*n* = 7) gastric cancer were included in the study. Samples were collected in the gastroenterology departments of four hospitals of the Mexican Institute of Social Security in Guadalajara, Mexico, during endoscopy for suspected malignant lesion. The samples were preserved in RNA later and frozen at − 20 °C, while the diagnosis for diffuse or mixed gastric cancer were confirmed by a pathologist; only the cases with diagnosis confirmed were included in the study. Tumor DNA was extracted with the Invisorb® Spin Tissue Mini Kit (StratecBiomedical) from tissue sections obtained by cryostat. When was possible, we analyzed constitutive DNA, which was extracted from peripheral blood leukocytes by the salting out method; this sample represents the normal DNA constitution present in all the somatic cells of the patient.

### Helicobacter pylori detection

*Helicobacter pylori* infection was determined in the tumor DNA by PCR through the Helicobacter pylori 520 kit (Sacace Biotechnologies©)^.^

### CDH1 mutation analysis

Somatic mutations were searched in the promoter region (865 bp) and the 16 exons of the *CDH1* gene, including splice sites and adjacent intronic regions. The sequencing reaction was performed through next generation sequencing (NGS) with the Roche 454/GS Junior platform (details in the Additional file 1). The reads were aligned to the reference sequence GRCh38-Chr16 using the BWA-MEM algorithm [7]. All the variants considered were represented in more than 50 reads and had a value > 100 on the Phred quality score.

The variants were corroborated through capillary sequencing with the Abi Prism 310 Genomic Sequencer, using the BigDye™ Terminator v3.1 Cycle Sequencing Kit (Thermo Fisher Scientific Inc.©). Bioinformatics analysis used to characterize the identified variants included three tools: PROMO v.3.0.2 [8, 9], Human Splicing Finder v.3.0 (HSF) [10] and Translate ExPASy [11].

### CDH1 loss of heterozygosity analysis

To detect allelic loss at the *CDH1* gene (locus 16q22.1) three microsatellite markers were used: D16S3025 at the 5′ end of the *CDH1* locus, and D16S496 and D16S3067 at the 3′ end of the *CDH1* locus [12]. Microsatellites were identified in those patients with both tumor and constitutive DNA samples (15 subjects) in a multiplex fluorescent PCR with primers previously described [4]. Fifty ng of DNA were used as template, and GeneScan™ 120 LIZ™ (Applied Biosystems™) was employed as size standard. The fragments were separated using the ABI PRISM® 310 Genetic Analyzer and the results were analyzed with Peak Scanner™ Software v1.0 (Applied Biosystems™). The LOH was calculated with the following formula: LOH index = (N1/N2)/(T1/T2), corresponding to peak areas of N1 = constitutive DNA allele 1; N2 = constitutive DNA allele 2; T1 = tumor DNA allele 1; and T2 = tumor DNA allele 2. LOH was considered when the LOH index was more than or less than 1.04 ± 0.13 for D16S3025, 1.0 ± 0.67 for D16S496, and 1.06 ± 0.11 for D16S3067 [4]. LOH-positive results were confirmed by repeated testing.

### CDH1 promoter methylation analysis

For the methylation analysis, 300 ng of tumor DNA were converted using the EpiTect Bisulfite Kit (Qiagen®), the analysis was performed in those samples with sufficient amount of tumor DNA (*n* = 17). DNA of SNU-1 cell line, which is constitutively methylated for the *CDH1* gene, was used as a positive methylation control. A 173-bp fragment in the promoter region of the *CDH1* gene, including 12 CpG sites, was amplified by PCR with specific primers for the converted DNA [12]. The PCR product was sequenced in the ABI 3130 Automated Capillary DNA sequencer (Applied Biosystems™). *CDH1* methylation in the gastric tumors was considered when at least 25% of the CpG sites analyzed showed methylation or hemi-methylation [12].

### E-cadherin expression

Immunohistochemistry was achieved in 16/20 cases (12 DGC and 4 MGC), frozen tissue sections of 5 μm were used for analysis with Liquid Mouse Monoclonal Antibody E-Cadherin (NCL-L-E-Cad, Leica™) according to supplier instructions. Tumor membranous staining for E-cadherin protein was scored using the following scale: 1+, low weak staining on the membrane; 2+, low to moderate staining on the membrane; 3+, moderate to strong staining on the membrane; and 4+ strong staining. Criteria defining E-cadherin deficiency were the scores 1+ or 2+; scores 3+ or 4+ were considered as normal expression.

### Compliance with ethical standards

The authors declare that they have no conflict of interest. Research involving human participants: all procedures performed in this study were in accordance with the ethical standards of the institutional and/or national research committee and with the 1964 Helsinki declaration and its later amendments or comparable ethical standards. Informed consent: written informed consent was obtained from all participants included in the study. This research was approved by the ethics committee of the Instituto Mexicano del Seguro Social.

## Results

### Characteristics of the samples analyzed

The sex ratio in all the patients with GC was 2.2:1 (male:female). When the cases were separated by histotype the ratio was 1.6:1 for DGC and 2.5:1 for MGC. Regarding age, the overall average was 62.2 years, while by histotypes the average was 60.8 and 71.2 years in DGC and MGC, respectively. Predominant blood types were A+ in the DGC group (4/11, 36.4%) and O+ in the MGC group (4/7, 57.1%). A family history of cancer, considered when at least one first-degree relative has been diagnosed with any type of cancer, was positive in the 57.1% of the MGC cases, while was only found at 15.4% in the DGC cases. *Helicobacter pylori* infection was detected in 58.3% [7/12] and 50% [3/6] of the diffuse and mixed gastric cancer cases, respectively (full information of each patient can be found in Additional file 2).

### CDH1 somatic variants observed through NGS

**Fig. 1:**
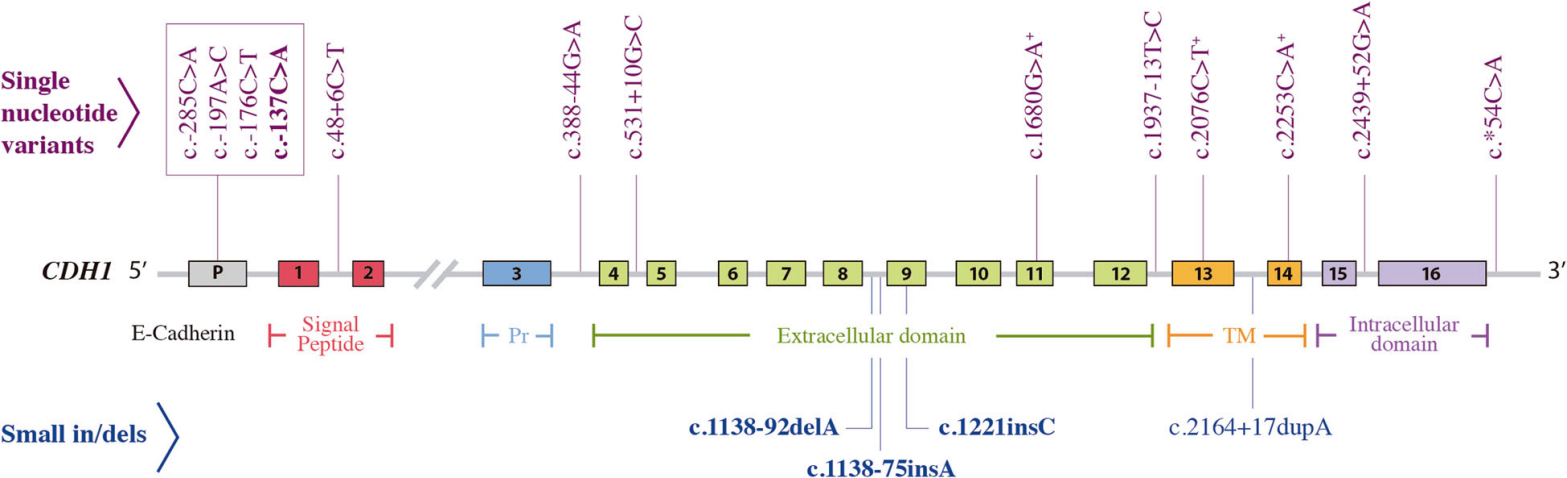
Type and localization of the variants found in the *CDH1* gene by next generation sequencing. The new variants are shown in bold; ^+^ Synonymous variants. P: promoter. Pr: precursor. TM: transmembrane region

A total of 17 variants were identified by NGS. Four of them were not previously described: c.-137C > A (located in the promoter region), c.1138-92delA, c.1138-75insA (intron 8) and c.1221insC (exon 9). The other 13 variants are known polymorphisms (Table 1). The variants were distributed throughout the gene, within the promoter (23.5%), introns (47.1%), exons (23.5%), and the 3’-UTR region (5.9%) (Fig. 1).

**Table 1:** Mutations and polymorphisms identified in the gastric cancer samples in the *CDH1* gene

New variants							
Location	Variant	Impact^a^	Heterozygous *n* (%)	Homozygous *n* (%)	DGC *n* (%)	MGC *n* (%)	
Promoter	c.-137C > A	Modifier	1 (5)	–	1 (5)	–	
Intron 8	c.1138-92delA	Modifier	3 (15)	–	3 (15)	–	
Intron 8	c.1138-75insA	Modifier	6 (30)	–	4 (20)	2 (10)	
Exon 9	c.1221insC	High	2 (10)	–	1 (5)	1 (5)	
Previously reported variants							
Location	Variant	Rs	ClinVar/Impact^a^	Heterozygous n (%)	Homozygous n (%)	DGC n (%)	MGC n (%)
Promoter	c.-285C > A /-160C > A	rs16260	Other/Modifier	4 (20)	1 (5)	3 (15)	2 (10)
Promoter	c.-197A > C /-73A > C	rs28372783	NI/Modifier	3 (15)	–	2 (10)	1 (5)
Promoter	c.-176C > T	rs34500817	NI/Modifier	1 (5)	–	1 (5)	–
Intron 1	c.48 + 6C > T	rs3743674	Benign/Low	9 (45)	10 (50)	13 (65)	7 (35)
Intron 3	c.388-44G > A	rs368884824	NI/Modifier	1 (5)	–	1 (5)	–
Intron 4	c.531 + 10G > C	rs33963999	Benign/Modifier	1 (5)	–	1 (5)	–
Exon 11	c.1680G > A	rs35741240	Benign/Low	1 (5)	–	1 (5)	–
Intron 12	c.1937-13 T > C	rs2276330	Benign/Modifier	2 (10)	1 (5)	–	3 (15)
Exon 13	c.2076C > T	rs1801552	Benign/Low	9 (45)	11 (55)	12 (60)	7 (35)
Intron 13	c.2164 + 17dupA	rs35686369	NI/Modifier	4 (20)	9 (45)	9 (45)	4 (20)
Exon 14	c.2253C > A	rs33964119	Benign/Low	4 (20)	–	3 (15)	1 (5)
Intron 15	c.2439 + 52G > A	rs33965115	NI/Modifier	–	1 (5)	–	1 (5)
UTR 3’	c.^a^54C > A	rs1801026	Benign/Modifier	5 (25)	–	4 (20)	1 (5)

^a^: According to the Variant Effect Predictor (Ensembl ®). *DGC* diffuse gastric cancer. *MGC* mixed gastric cancer. *Rs* reference SNP. *NI* No information

### CDH1 new variants

**Fig. 2:**
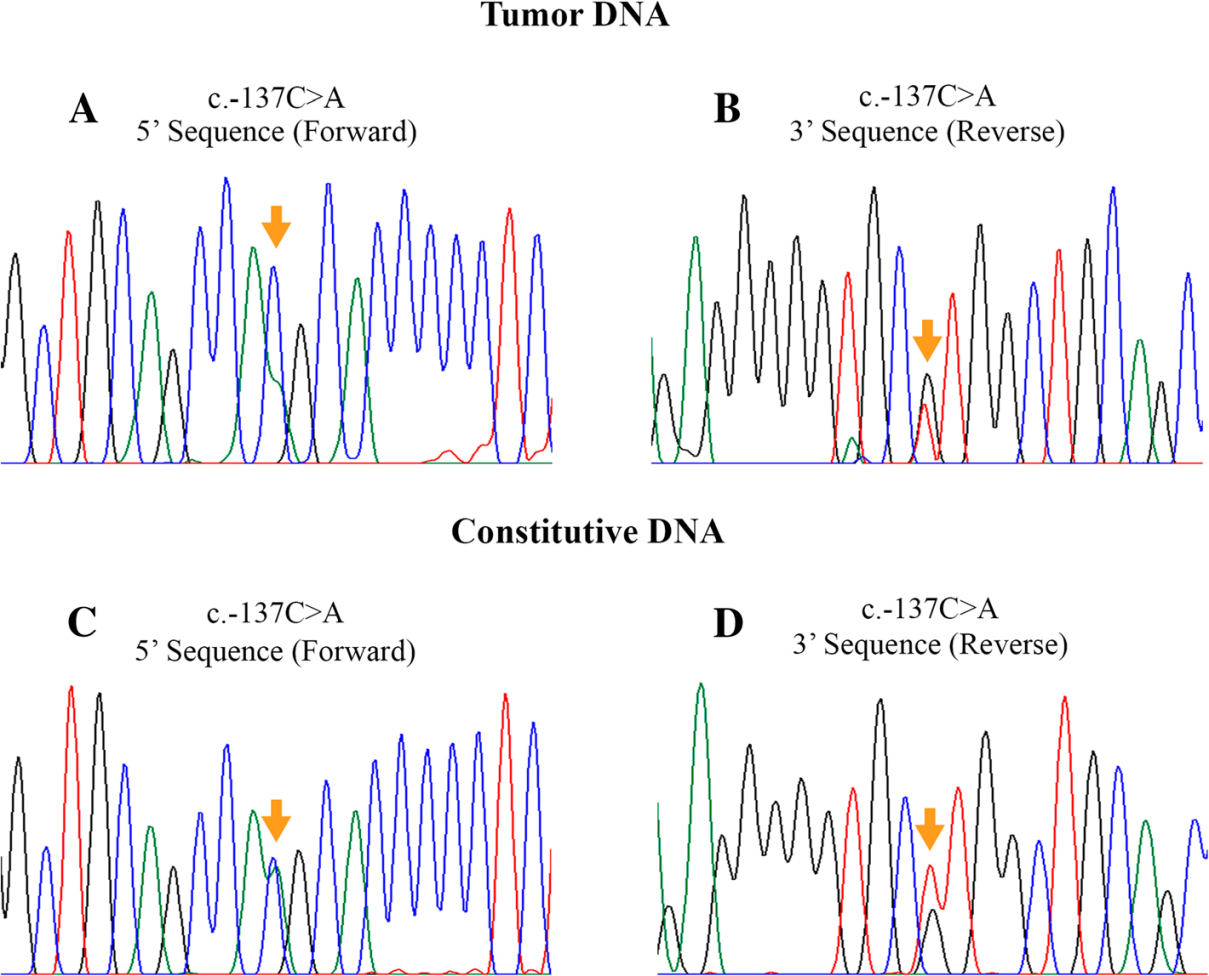
Sanger sequencing of the c.-137C > A variant. **a**) and **b**) show the results in the tumor DNA, confirmed in the constitutive DNA (**c** and **d** images). The mutation site is indicated (arrows)

From the four new variants, only c.-137C > A was validated through Sanger sequencing in the tumor and constitutive DNA in heterozygous state (Fig. 2); the PROMO software predicted that this transversion creates novel binding sites for transcription factors (NFI/CTF and C/EBPbeta). To investigate if the c.-137C > A variant is a polymorphism in the healthy Mexican population, we analyzed 98 genomic DNA samples from blood donors, and all samples were negative for the variant. This shows that the c.-137C > A variant is absent in our population. The variants c.1138-92delA, c.1138-75insA (intron 8) and c.1221insC (exon 9) were not validated by Sanger sequencing method because the results did not reveal their presence both in genomic and tumor DNA, in other words, sequences were normal (wild alleles).

### CDH1 known variants

All known variants found by NGS were validated by Sanger sequencing, except the c.388-44G > A. According their ClinVar report and the Variant Effect Predictor (VEP) tool, the variant c.-285C > A was classified as “other/modifier” respectively, four variants (c.48 + 6C > T, c.1680G > A, c.2076C > T, and c.2253C > A) were reported as “benign/low”, four variants (c.531 + 10G > C, c.1937-13 T > C, c.2164 + 17dupA, and c.*54C > A) as “benign/modifier”, and three variants (c.-197A > C, c.-176C > T, c.2439 + 52G > A) only had VEP information available and were predicted to have a “modifier” effect.

In silico analysis was performed for the variants (c.-197A > C, c.-176C > T, c.2164 + 17dupA, and c.2439 + 52G > A). The results showed that the variants c.-197A > C (rs28372783) and c.-176C > T (rs34500817) modify the binding sites for some transcription factors; specifically, the c.-197C allele was found to create two additional transcription factor (ER-alpha and C-jun) binding sites and to delete four (VDR, FOXP3, RAR-beta, PXR-1; with the c.-176 T allele, a new binding site for the transcription factor YY1 is created. Regarding the variants c.2164 + 17dupA and c.2439 + 52G > A, found in introns 13 and 15 respectively, the HSF tool shows that both c.2164 + 15A and c.2439 + 52A alleles alters the exonic splicing silencer (ESS) site and an exonic splicing enhancer (ESE) site is created.

### CDH1 loss of heterozygosity

**Fig. 3:**
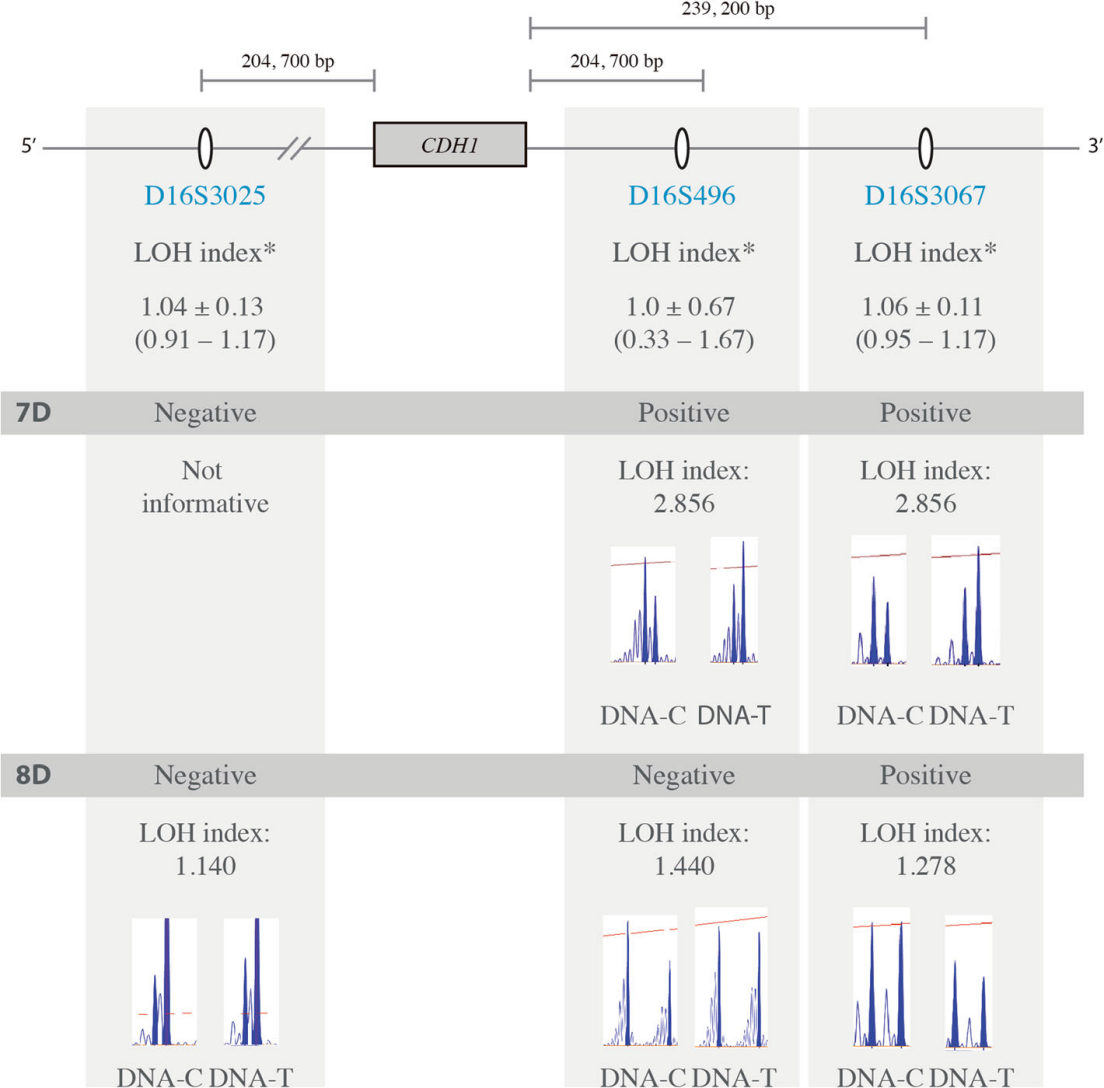
Representative image of the microsatellite markers used for the loss of heterozygosity analysis. The two LOH positive cases are shown. The formula employed was: LOH index = (N1/N2)/(T1/T2), corresponding to peak areas of N1 = constitutive DNA allele 1; N2 = constitutive DNA allele 2; T1 = tumor DNA allele 1; and T2 = tumor DNA allele 2 [4]. The distance of each marker from the *CDH1 locus* is approximate

The LOH analysis was carried out for 15/20 patients, for whom tumor and constitutive DNA were available. All the samples had at least two informative markers. Two DGC cases (13.3%) showed LOH: the 7D sample presented LOH at the D16S496 and D16S3067 markers, located at the 3′ end of the *CDH1 locus*, whereas the 8D sample showed LOH only for the microsatellite D16S3067 (Fig. 3) (Full microsatellites genotypes can be consulted in the Additional file 3).

### CDH1 methylation

**Fig. 4:**
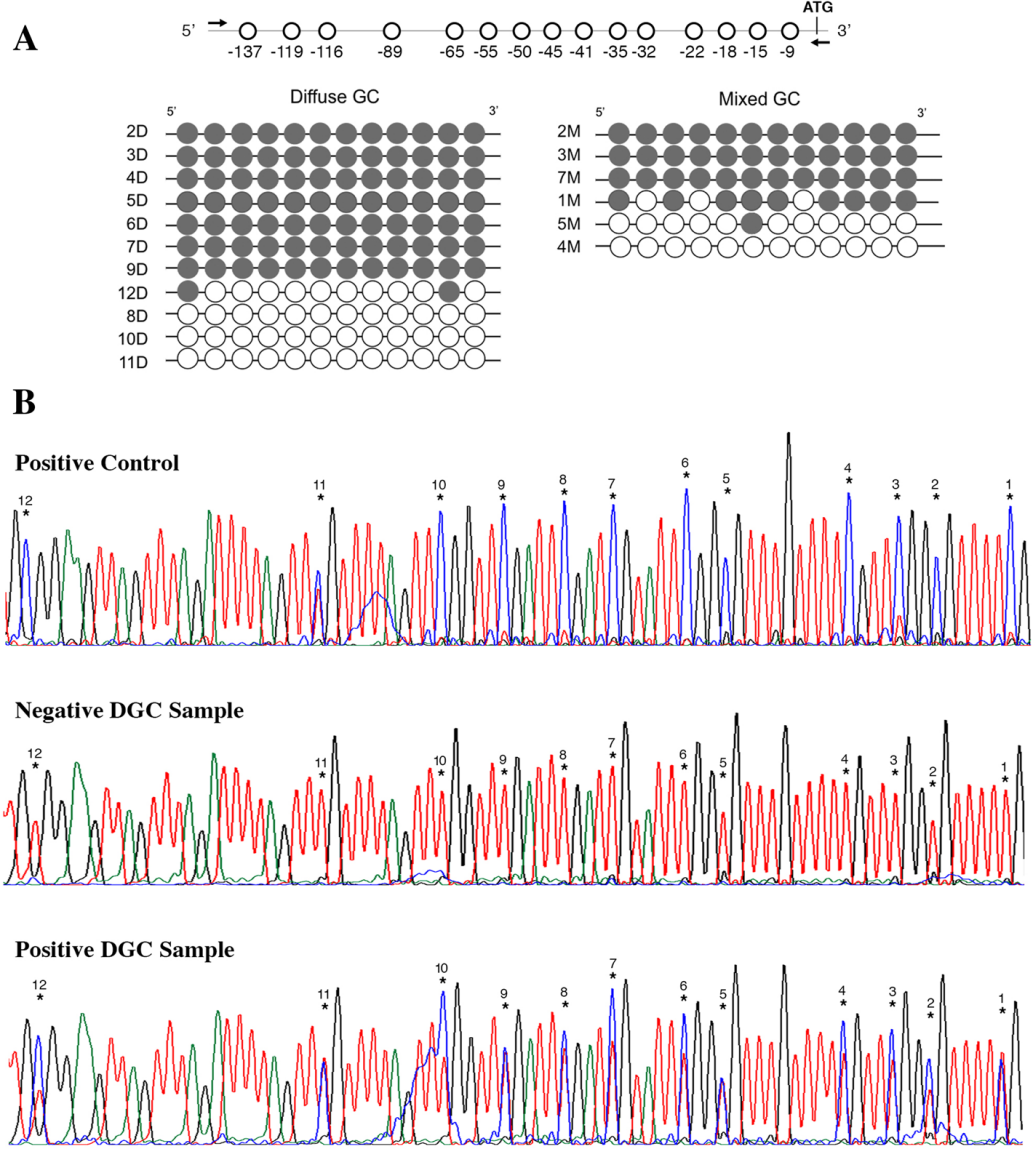
Methylation analysis results. **a**) Representation of the promoter region analyzed for methylation. Filled circles represent the methylated CpG sites and clear circles symbolize non-methylated sites. Each line represents one individual (ID shown). **b**) Sequencing results for the methylation analysis. The CpG sites are numbered

In 17/20 GC samples (DGC *n* = 11 and MGC *n* = 6), the methylation pattern in the *CDH1* gene promoter was analyzed. The results showed 11/17 cases that were positive for methylation (64.7%), seven of which were DGC (63.3% in DGC cases) and four of which MGC (66.7% in MGC cases) (Fig. 4). From the patients with *CDH1* methylation, 63.6% were also positive for *H. pylori*; nevertheless, this association was not significant (*p* > 0.05).

### E-cadherin expression

Immunohistochemistry results revealed E-cadherin protein deficiency, low (+) to moderate (++) weak, in 58.3% (7/12) of DGC cases while 100% (4/4) in MGC cases.

The relation of *CDH1* methylation and low expression of E-cadherin was also analyzed, we observed that 85.7% (6/7) of *CDH1* methylated DGC cases had deficiency of E-cadherin (IHQ + or ++), whereas for the methylated MGC cases, all of them (100% 3/3) had E-cadherin deficiency.

## Discussion

*CDH1* is the main affected gene in DGC, both at the somatic and germline levels. In this study, the somatic genetic and epigenetic alterations in *CDH1* were characterized in Mexican patients with DGC and MGC.

### CDH1 somatic variants

*CDH1* mutations have been reported with frequencies ranging from 4.5 to 50% in diffuse tumors [4, 5]. In our study, a total of 13 validated variants (including polymorphisms) in the *CDH1* gene were observed; however, we have not evidence to consider any of them as pathogenic.

The c.-137C > A variant found was previously reported for the Human Longevity Project (rs1046078040) [13] but frequency and functionality data is lacking. In silico analysis suggested that this variant can affect the expression of E-cadherin protein, because two new binding sites for the transcription factors NFI/CTF and C/EBPbeta are created, as well the binding site for the transcription factor AhR: Arnt destroyed. Immunohistochemistry confirm the low expression of E-cadherin in the carrier of the c.-137C > A variant. Dysregulation in the expression of the *CDH1* gene can be explained by the modification of the binding sites for transcription factors, the NFI transcription factor play an important role during normal development, it functions by regulating cell proliferation and differentiation via the transcriptional control of their target genes, also it has been implicated in cancer, evidences suggests a converging role in development and cancer, with both oncogenic and tumor suppressor potential, depending on the carcinoma type and its tissue origin (Chen et al., 2017 [14]). On the other hand, C/EBPbeta activates transcription of genes with a specific role in the nervous system, cytokines and transporter proteins that confer multidrug resistance as ABCC2 and ABCB1. C/EPBbeta is overexpressed in gastric tumors, with notable differences between histotypes, MGC (50%), IGC (46.2%) and DGC (4.4%) [15]). Regarding to the AhR, it have a role in TCDD toxicity, but also is involved in tumorigenesis and is found at elevated levels in aggressive tumors and tumor cell lines [16], however with the variant the binding site for this transcription factor is destroyed.

Regarding the variants c.1138-92delA, c.1138-75insA, and c.1221insC detected by NGS but not confirmed by capillary sequencing, it is important to highlight the need to confirm NGS findings as false positives can occur, particularly in repeated mononucleotides regions [17]; another possibility is that, due to NGS higher sensitivity, such variants could be detected even if is present in a minority of DNA molecules within the tumor. Regarding the c.1221insC variant, interestingly, a mutation affecting neighbor nucleotides, c.1220delC (p.P407Qfs10), was reported in three members of a Spanish family with hereditary DGC [18]; both, the c.1221insC variant and the c.1220delC mutation are predicted to result in a premature stop codon, which would lead to mRNA degradation and therefore to reduced E-cadherin expression and carcinogenesis, however c.1221insC was not confirmed through Sanger sequencing method.

Regarding known variants c.-197A > C, c.-176C > T, c.2164 + 17dupA, and c.2439 + 52G > A, in silico analysis showed that those located within the promoter can lead to changes in the binding site of some transcription factors. Recently was reported that the variant c.-197A > C (also denominated -73A > C) may lead to allele-specific repressions of *CDH1* gene, the allele C was related to lesser methylation, higher transcription levels and longer survival [19], in our study observed three carriers for this allele, only one of them had normal expression of E-cadherin protein and CDH1 no methylated. For the c.-176C > T variant, no information published about their impact or functionality on CDH1 gene expression. The variants c.2164 + 17dupA and c.2439 + 52G > A, alters exonic splicing sites (ESS and ESE), the c.2164 + 17dupA is a common non-coding variant observed in 13/20 patients in our study, it is considered as benign by ClinVar, also, it was found in patients with breast cancer but was considered as no pathogenic [20]). On the other hand, the c.2439 + 52G > A variant was observed in our study only in one patient, the clinical significance information is not yet reported in ClinVar, so in both cases the functional role need be clarified in future research.

For the known variants c.-285C > A (rs16260), c.48 + 6C > T (rs3743674), c.531 + 10G > C (rs33963999), c.1680G > A (rs35741240), c.1937-13 T > C (rs2276330), c.2076C > T (rs1801552), c.2164 + 17dupA (rs35686369), c.2253C > A (rs33964119), and c.54C > A (rs1801026), ClinVar considers them all as variants with clinical significance “benign” except for c.-285C > A that consider it as “risk factor” for prostate cancer. On the other hand, the Variant Effect Predictor tool predicts a “low” impact for the variants c.48 + 6C > T, c.1680G > A, c.2076C > T, and c.2253C > A, while the variants c.-285C > A, c.531 + 10G > C, c.1937-13 T > C, and c.54C > A are considered with an impact “modifier”. Additionally, some of these variants (rs35741240, rs1801552, rs33964119) are located in exonic regions but they are synonymous variants; others are located in introns or in non-translated regions (rs3743674, rs33963999, rs2276330, rs1801026) but to date no effect on the transcription process has been reported.

An important aspect to consider is that, with the exception of variant -285C > A (or -160C > A) [21], no other variants have been described for the Mexican population. It would be important to investigate the frequencies of the variants described in this population (Additional file 4).

### CDH1 loss of heterozygosity

LOH on specific chromosomal regions is related to the inactivation of tumor suppressor genes. In fact, focal genomic deletions in tumor suppressor genes such as *PTEN, SMAD4, PARK2, RB1, CDKN2A,* and *ARID1A* have been reported in GC [22–24]. Importantly, allelic loss in the 16q22.1 *locus* is a genetic mechanism implicated in the inactivation of the *CDH1* gene; up to 91% of the GC cases with LOH in that locus present a reduced expression of E-cadherin [25]. In our study, the frequency of LOH in 16q22.1 was 13.3%, corresponding to two DGC cases, which is in accordance with ranges reported between 4.5 and 39% [4, 25, 26]. In both patients, we assume that the LOH found does not correspond to intragenic deletions as it involves only the 3′ of the gene’s *locus*. This is corroborated by the fact that some of the variants identified by NGS, for these patients, were observed in heterozygote status (Additional file 2). Additionally, both samples were also analyzed by Multiplex Ligation-dependent Probe Amplification (MLPA) in the tumor DNA (probe SALSA P083-C2 CDH1, MRC Holland) and no major *CDH1* deletions were found.

### CDH1 methylation

Methylation of the *CDH1* promoter is the most common epigenetic modification associated with the loss of heterozygosity in DGC cases. It also has been reported that 82% of cases with methylation have reduced expression of E-cadherin [25]. The frequency of *CDH1* methylation in GC patients has been reported in 25.4 to 76% in Asian and Caucasian populations [4, 25, 27]. In this study, we observed a frequency of 64.7% (*n* = 11 cases) and non-significant differences were observed regarding the gastric cancer histotype. Further, *H. pylori* infection has been strongly associated with DNA methylation, specifically with the *CDH1* gene [28]; in our study, DGC cases positives for H pylori infection, 57.1% of them had *CDH1* promoter methylation and E-cadherin deficiency, and of the MGC cases positives for H pylori, 66.6% had both methylation and E-cadherin deficiency.

### Multiple CDH1 alterations

More than one second hit mechanism has been reported in patients with hereditary DGC [12]. In our study, one DGC patient (men of 57 years old) was carrier of three alterations in the *CDH1* gene: methylation, LOH and the new variant c.-137C > A, although it has no family history of GC. Most probably the finding of concomitant somatic inactivating mechanisms results from intratumor heterogeneity.

Finally, although the small number of samples and the absence of histopathological images constitute limitations to our study, the results it provides, regarding the alterations in the *CDH1* gene and its importance for the Mexican population with diffuse and mixed gastric cancer, sheds light on a topic which has not been studied so far.

## Conclusions

We characterized, for the first time, the landscape of somatic genetic and epigenetic alterations in *CDH1* for Mexican patients with DGC and MGC and observed that the main inactivating events were promoter methylation and LOH. Pathogenic mutations were not found; however, a new variant in the promoter of the *CDH1* gene (− 137 C > A) was observed, which could result in E-cadherin expression downregulation. Further studies are warranted to address its functionality.

## Additional files

Additional file 1: CDH1 NGS mutation analysis. Contains additional information about the methodology followed in the next generation sequencing process. (PDF 50 kb)

Additional file 2: Characteristics and alterations found in all the patients. Contains a complete table with the description of all the somatic variants presented by each patient in the study. (PDF 89 kb)

Additional file 3: Genotypes of the microsatellites markers used for the LOH analysis. Contains complete genotypes of the microsatellites markers used for the LOH analysis. (PDF 50 kb)

Additional file 4: SNP frequencies in Hispanic populations. Contains frequencies in other Hispanic populations, of the polymorphisms found in this study. (PDF 67 kb)

## Abbreviations

DGC: Diffuse Gastric Cancer
DNA: Deoxyribonucleic Acid
GC: Gastric Cancer
HSF: Human Splicing Finder v.3.0
LOH: Loss of Heterozygosity
MGC: Mixed Gastric Cancer
MLPA: Multiplex Ligation-dependent Probe Amplification
mRNA: Messenger RNA
NGS: Next Generation Sequencing
PCR: Polymerase Chain Reaction
RNA: Ribonucleic Acid
VEP: Variant Effect Predictor
