# Supplementary material for: CDH1 somatic alterations in Mexican patients with diffuse and mixed sporadic gastric cancer
Source: BMC Cancer. 2019 Jan 14;19:69. doi: 10.1186/s12885-019-5294-0 (PMC6332846; doi:10.1186/s12885-019-5294-0)
Supplement: Supplementary file 1 — CDH1 NGS mutation analysis. Contains additional information about the methodology followed in the next generation sequencing process. (PDF 50 kb) [file 12885_2019_5294_MOESM1_ESM.pdf]

Additional file 1

Methods

CDH1 NGS mutation analysis

To create the NGS library, a first round of PCR was done with universal and specific primers for each fragment (some primers previously described by Corso et al., 2013, and some newly designed; sequences available upon request). The PCR products were corroborated by polyacrylamide electrophoresis, purified with AMPure beads (AgencourtAMPure XP BeckmanCoulter, Inc.®) and quantified using the fluorometer Qubit vs. 3.0® (Life Technologies®) with the dsDNA BR and dsDNA HS Assay Kits (Life Technologies®).

All the fragments of each patient were mixed in a single tube to add a specific identifier sequence (barcode) in a second round of PCR. The same protocol for purification was followed, and a proper confirmation by electrophoresis in chip was done with the Agilent 2100 Bioanalyzer® and the kit Agilent DNA 7500 Assay®. All samples were quantified as previously described, and diluted to equimolar concentrations. Finally, the barcoded samples of all the patients were mixed in a single final stock and adjusted to $2x10^6$ molecules/mL. With this DNA-work solution, emulsion PCR (emPCR) was performed following the provider's standard protocol. The kits used were the GS Junior Titanium emPCR kit Lib A for the emPCR, the GS Junior Titanium Sequencing Kit and the GS Junior Titanium PicoTiterPlate Kit for the sequencing reaction and plate loading, respectively. The reaction was run in the GS Junior Sequencer. Roche Diagnostics provided all the kits used.
