# Supplementary material for: CDH1 somatic alterations in Mexican patients with diffuse and mixed sporadic gastric cancer
Source: BMC Cancer. 2019 Jan 14;19:69. doi: 10.1186/s12885-019-5294-0 (PMC6332846; doi:10.1186/s12885-019-5294-0)
Supplement: Supplementary file 2 — Characteristics and alterations found in all the patients. Contains a complete table with the description of all the somatic variants presented by each patient in the study. (PDF 89 kb) [file 12885_2019_5294_MOESM2_ESM.pdf]

Additional file 2

Table S1. Characteristics and alterations found in all the patients.

DIFFUSE GASTRIC CANCER CASES									
**ID**	**Sex**	**Age**	**Hemotype**	***H. pylori***	**Family history**	**Variants**	**Geno-type**	**LOH**	**Methyl.**
1D	M	68	A+	-	No	c.1138-75insA<br>c.2076T>C<br>c.48+6C>T	in/del<br>C/C<br>T/T	NA	NA
2D	M	57	A-	+	No	c.2076T>C	C/C	-	+
3D	M	38	NI	+	No	c.-197A>C / c.-73A>C<br>c.48+6C>T<br>c.2076T>C<br>c.2164+17dupA<br>c.*54C>A	A/C<br>C/T<br>T/C<br>dupA/dupA<br>C/A	-	+
4D	M	54	O+	+	No	c.48+6C>T<br>c.1138-75insA<br>c.2076T>C	C/T<br>in/del<br>C/C	-	+
5D	M	63	A+	+	No	c.-160C>A / c.-285C>A<br>c.48+6C>T<br>c.2076T>C	A/A<br>T/T<br>T/C	-	+
6D	F	86	A+	-	Yes (1st degree)	c.48+6C>T<br>c.531+10G>C<br>c.1138-75insA<br>c.2076T>C<br>c.2164+17dupA	C/T<br>G/C<br>in/del<br>T/C<br>dupA/dupA	-	+
7D	M	57	A-	-	No	c.-137C>A<br>c.48+6C>T	C/A<br>T/T	LOH +	+

						c.388-44G>A	G/A		
						c.2076T>C	T/C		
						c.2164+17dupA	dupA/dupA		
8D	M	70	B+	+	Yes (1st degree)	c.-176C>T	C/T	LOH Border-line	-
						c.48+6C>T	C/T		
						c.1138-75insA	ins/del		
						c.1680G>A	G/A		
						c.2076T>C	C/C		
						c.2164+17dupA	del/dupA		
						c.2253C>A	C/A		
						c.*54C>A	C/A		
9D	F	64	NI	-	No	c.48+6C>T	T/T	-	+
						c.1221insC	ins/del		
						c.2076T>C	T/C		
						c.2164+17dupA	dupA/dupA		
10D	M	74	B+	+	No	c.-160C>A / c.-285C>A	C/A	-	-
						c.48+6C>T	C/T		
						c.2076T>C	C/C		
						c.2164+17dupA	dupA/dupA		
11D	F	53	A+	+	Yes (2nd degree)	c.48+6C>T	T/T	-	-
						c.2076T>C	C/C		
						c.2164+17dupA	del/dupA		
						c.2253C>A	C/A		
						c.*54C>A	C/A		
12D	F	45	O+	-	No	c.-160C>A / c.-285C>A	C/A	NA	-
						c.-197A>C / c.-73A>C	A/C		
						c.48+6C>T	T/T		
						c.1138-92delA	ins/del		

						c.2076T>C<br>c.2164+17dupA<br>c.2253C>A<br>c.*54C>A	C/C<br>del/dupA<br>C/A<br>C/A		
13D	F	53	O+	NA	Yes (2nd degree)	c.48+6C>T<br>c.1138-92delA<br>c.2076T>C<br>c.2164+17dupA	T/T<br>ins/del<br>C/C<br>dupA/dupA	NA	NA
Total (n=13)	M: 61.5%<br>F: 38.5%	60.75 (30-86)	A+: 36.4%<br>A-: 18.2%<br>B+: 18.2%<br>O+: 27.2%	(+): 58.3%<br>(-): 41.7%	1st d.: 15.4%<br>2nd d.: 15.4%			13.3%	63.6%

MIXED GASTRIC CANCER CASES

ID	Sex	Age	Hemotype	*H. pylori*	Family history	Variants	Genotype	Allelic Loss	Methyl.
1M	M	79	A+	+	Yes (1st degree)	c.48+6C>T<br>c.2076T>C	T/T<br>T/C	-	+
2M	M	72	O+	-	Yes (2nd degree)	c.48+6C>T<br>c.1138-75insA<br>c.2076T>C<br>c.2164+17dupA	C/T<br>in/del<br>C/C<br>dupA/dupA	-	+
3M	F	58	B+	+	Yes (1st degree)	c.48+6C>T<br>c.1138-75insA<br>c.1937-13T>C<br>c.2076T>C<br>c.2164+17dupA	C/T<br>in/del<br>T/C<br>T/C<br>dupA/dupA	-	+
4M	F	62	O+	-	Yes (1st degree)	c.48+6C>T<br>c.2076T>C<br>c.2439+52G>A	T/T<br>C/C<br>A/A	-	-
5M	M	NI	O+	+	NI	c.48+6C>T	C/T	NA	-

						c.-197A>C / c.-73A>C	A/C		
						c.2076T>C	T/C		
						c.2164+17dupA	del/dupA		
						c.2253C>A	C/A		
						c.*54C>A	C/A		
6M	M	78	O+	NR	Yes (1st degree)	c.48+6C>T	T/T	NA	NA
						c.-160C>A / c.-285C>A	C/A		
						c.1221insC	ins/del		
						c.1937-13T>C	T/C		
						c.2076T>C	T/C		
						c.2164+17dupA	dupA/dupA		
7M	M	78	A+	-	No	c.48+6C>T	C/T	-	+
						-160C>A / c.-285C>A	C/A		
						c.1138-92delA	ins/del		
						c.1937-13T>C	C/C		
						c.2076T>C	C/C		
Total (n=7)	M: 71.4%<br>F: 28.6%	71.2 (58-79)	A+: 28.6%<br>B+: 14.3%<br>O+: 57.1%	(+) 50%<br>(-) 50%	1st d.: 57.1%<br>2nd d.: 14.3%			0%	36.4%

LOH: loss of heterozygosity; NA: not analyzed;
