# Supplementary material for: CDH1 somatic alterations in Mexican patients with diffuse and mixed sporadic gastric cancer
Source: BMC Cancer. 2019 Jan 14;19:69. doi: 10.1186/s12885-019-5294-0 (PMC6332846; doi:10.1186/s12885-019-5294-0)
Supplement: Supplementary file 3 — Genotypes of the microsatellites markers used for the LOH analysis. Contains complete genotypes of the microsatellites markers used for the LOH analysis. (PDF 50 kb) [file 12885_2019_5294_MOESM3_ESM.pdf]

Additional file 3

Table S2. Genotypes of the microsatellites markers used for the LOH analysis.

DIFFUSE GASTRIC CANCER				
**Sample**	**D16S3025**	**D16S496**	**D16S3067**	**LOH**
2D-C	97/101	218/222	148/150	No
2D-T	97/101	218/222	148/150	
3D-C	99/101	209/220	148/154	No
3D-T	99/101	209/220	148/154	
4D-C	95/101	221/223	148/154	No
4D-T	95/101	221/223	148/154	
5D-C	103/103	218/220	146/152	No
5D-T	103/103	218/220	146/152	
6D-C	97/101	209/218	144/150	No
6D-T	97/101	209/218	144/150	
7D-C	88/88	220/222	150/152	LOH
7D-T	87/102	219/221	150/152	
8D-C	101/101	210/221	148/152	LOH
8D-T	101/101	210/221	148/152	
9D-C	99/101	220/220	148/152	No
9D-T	99/101	220/220	148/152	
10D-C	101/103	219/221	148/152	No
10D-T	101/103	219/221	148/152	
11D-C	101/103	209/220	146/148	No
11D-T	101/103	209/220	146/148	
**MIXED GASTRIC CANCER**				
**Sample**	**D16S3025**	**D16S496**	**D16S3067**	**LOH**
1M-C	95/102	222/224	150/152	No
1M-T	95/102	222/224	150/152	
2M-C	99/101	220/220	152/154	No
2M-T	99/101	220/220	152/154	
3M-C	101/103	142/148	210/221	No
3M-T	101/103	142/148	210/221	
4M-C	101/102	218/220	144/152	No
4M-T	101/102	218/220	144/152	
7M-C	87/104	210/223	152/154	
7M-T	87/104	210/223	152/154	No
