# Supplementary material for: CDH1 somatic alterations in Mexican patients with diffuse and mixed sporadic gastric cancer
Source: BMC Cancer. 2019 Jan 14;19:69. doi: 10.1186/s12885-019-5294-0 (PMC6332846; doi:10.1186/s12885-019-5294-0)
Supplement: Supplementary file 4 — SNP frequencies in Hispanic populations. Contains frequencies in other Hispanic populations, of the polymorphisms found in this study. (PDF 67 kb) [file 12885_2019_5294_MOESM4_ESM.pdf]

Additional file 4

Table S3. SNP frequencies in Hispanic populations.

rs	2n	Alt. Alelle (IG)	Frequency in Hispanics (IG Source)			2n	Alt. Alelle (AF)	Frecuency in Hispanics (AF Source)			Present study*
**rs16260**	100	A: 0.280 [a]	C/C: 0.620[a]	C/A: 0.300 [a]	A/A: 0.080 [a]	186	A: 0.280 [b]	C/C: 0.510 [b]	C/A: 0.430 [b]	A/A: 0.070 [b]	A: 0.125
**rs28372783**	44	C: 0.068	A/A: 0.864	A/C: 0.136	C/C: 0.000	42	C: 0.071	A/A: 0.904	A/C: 0.048	C/C: 0.047	A: 0.075
**rs34500817**	44	T: 0.000	C/C: 1.000	C/T: 0.000	T/T: 0.000		T: -	-	-	-	T: 0.025
**rs3743674**	44	T: 0.727	C/C: 0.136	C/T: 0.273	T/T: 0.591	694 [c]	T: 0.776[c]	-	-	-	T: 0.675
**rs33932809**	44	T: 0.023	C/C: 0.954	C/T: 0.045	T/T: 0.000	46	T: 0.000	C/C: 1.000	C/T: 0.000	T/T: 0.000	T: 0.025
**rs368884824**		-	-	-	-	694 [c]	A: 0.001 [c]	-	-	-	A: 0.025
**rs33963999**	42	C: 0.000	G/G: 1.000	G/C: 0.000	C/C: 0.000	46	C: 0.000	G/G: 0.956	G/C: 0.043	C/C: 0.000	C: 0.025
**rs35741240**	44	C: 0.000	G/G: 1.000	G/C: 0.000	C/C: 0.000		-	-	-	-	C: 0.025
**rs2276330**	100[a]	C: 0.090 [a]	T/T: 0.860 [a]	T/C: 0.100 [a]	C/C: 0.040 [a]	44	C: 0.023	T/T: 0.954	T/C: 0.045	C/C: 0.000	C: 0.100
**rs1801552**	100 [a]	T: 0.310 [a]	C/C: 0.479[a]	T/C: 0.419 [a]	T/T: 0.101 [a]	46	T: 0.435	C/C: 0.304	T/C: 0.522	T/T: 0.174	T: 0.725
**rs35686369**		-	-	-	-	46	A: 0.065	-/-: 0.913	-/+: 0.043	+/+: 0.043	A: 0.5
**rs33964119**	44	T: 0.114	C/C: 0.773	C/T: 0.227	T/T: 0.00	44	T: 0.091	C/C: 0.864	C/T: 0.091	T/T: 0.045	T: 0.1
**rs33965115**	42	A: 0.023	G/G: 0.952	G/A: 0.047	A/A: 0.00	46	A: 0.065	G/G: 0.870	G/A: 0.130	A/A: 0.00	A: 0.05
**rs1801026**	42	T: 0.214	C/C: 0.571	C/T: 0.428	T/T: 0.00	45	T: 0.195	C/C: 0.695	C/T: 0.217	T/T: 0.087	T: 0.125

* Frequency of the alternative allele in DGC and MGC patients (2n=40). [a]HAPMAP-MEX. [b]Frequency reported in mexican population (Bustos-Carpinteyro, 2016) [18]. [C] AMR Population AMR: American.
